# Diet Biofunctionality in Modulating Cardiovascular Parameters in Obese Patients After Bioenteric Intragastric Balloon Bariatric Surgery

**DOI:** 10.3390/nu16234038

**Published:** 2024-11-26

**Authors:** Edyta Barbara Balejko, Jarosław Lichota

**Affiliations:** 1 Department of Commodity Sciences, Quality Assessment, Process Engineering and Human Nutrition, Faculty of Food Sciences and Fisheries, West Pomeranian University of Technology in Szczecin, 70-310 Szczecin, Poland; 2 Unii Lubelskiej 1, Department of General, Minimally Invasive and Gastroenterological Surgery, Independent Public Clinical Hospital No. 1 of Pomeranian Medical University in Szczecin, 71-252 Szczecin, Poland

**Keywords:** obesity, bariatric, dietotherapy, isoprostanes, plasminogen activator inhitor-1, angitensin, prostacyclins, triglycerides, hypertension, blood rheology

## Abstract

Background/Objectives: Adiposopathy is the cause of many secondary disorders in the function and structure of many organs and systems in the body. In the progression of obesity and the increasing dysfunction of anti-inflammatory me-diators, chronic inflammation occurs. This may be accompanied by the development of metabolic complications. Methods: A diet with highly bioactive properties was formulated, and an element of diet therapy was introduced as a key component to support treatment in obese patients following bariatric surgery. Patients underwent a BIB (Bioenteric Intragastric Balloon) procedure. As obesity is a risk factor for cardiovascular diseases, this study aimed to regulate cardiovascular factors in adiposopathy. Anti-inflammatory dietary components with modulating properties were included, with increased bioflavonoids, vitamins A, E, C, folic acid, and synbiotics, and altered fatty acid composition. Results: The results showed satisfactory effects on fat reduction and the regulation of isoprostanes, plasminogen, angiotensin, prostacyclin, triglycerides, blood pressure, and blood rheology parameters. Cardiovascular co-morbidities are common in obesity. This is due to the endocrine function of adipocytes. As such, we decided to investigate the possibility of using bioactive dietary components as an adjunct to the safety of reducing prothrombotic parameters in obese patients after BIB surgery. This study hypothesised that the enrichment of the recommended diet after bariatric surgery with the addition of *n*-3 EFAs, bioflavonoids, vitamins, and synbiotics might result in comparable or greater fat mass loss in the subjects. In addition, the use of a functional diet might show a beneficial modifying effect on antihyperglycemic parameter values. We then compared the results to those obtained among patients fed a reducing, standard diet. Conclusions: Using a functional diet, a significant reduction in visceral fat was achieved. A decrease in VFA was shown to reduce whole-blood viscosity. Furthermore, this study showed a significant effect of bioactive components on pro-thrombotic parameters in obese patients.

## 1. Introduction

The development of civilisation has greatly contributed to the extension of human life. At the same time, modern lifestyles have exacerbated the problem of obesity to epidemic proportions. Visceral fat dysfunction results from inflammation, oxidative stress, hypoxia, and mitochondrial dysfunction. The increased chemotaxis of immune cells into adipose tissue leads to the development of a chronic inflammatory process. With obesity come dysfunctions in blood volume and flow, systolic cardiac output, minute volume, cardiac hypertrophy, hypertension, and haemorheological properties. Due to interdependent metabolic disturbances, vascular wall damage, thrombus formation, and atherosclerotic plaques occur [1]. Most adipokines from visceral adipose tissue are pro-inflammatory. Adiposopathy is the cause of many secondary disorders in the function and structure of many organs and systems in the body. Adipokines entering the circulation exert effects on the function of the vascular walls of circulation and heart muscles. Atherosclerotic plaques in obese patients contain more lipids, are brittle, and easily rupture [2]. In the progression of obesity and the increasing dysfunction of anti-inflammatory mediators, chronic inflammation occurs. This may be accompanied by the development of metabolic complications. This fact explains the association of co-morbidities such as diabetes with an increased risk of inflammatory diseases, hypertension, and atherosclerosis. The exceptional incidence of cardiovascular complications prompts the search for support for therapeutic management. The functional properties of nutrients offer opportunities for the natural modulation of concentrations of factors that cause metabolic disorders [3,4,5]. Current diet therapy is based on designer foods that meet specific needs of the body. Long-term reduction diets very often lead to deficiencies. Therefore, this paper proposes a greater supply of bioactive natural ingredients such as bioflavonoids, fatty acids, and probiotics, with the expectation of significantly better treatment effects. A diet with functional characteristics offers the possibility of prevention and effective care. Therefore, the enrichment of weight-loss diets with functional food components may be appropriate.

For obese patients with BMI values ≥ 35 kg/m^2^, experiencing no effects in terms of weight reduction by conservative methods, bariatric surgery and procedures are used [6,7,8]. An intragastric balloon is the least invasive bariatric procedure. The aim of this study was to modify the recommended standard reduction diet after bariatric surgery to achieve the regulation of isoprostanes, plasminogen, angiotensin, prostacyclin, triglycerides, blood pressure, and blood rheological parameters. The regulation of the concentrations of these parameters was expected to increase the health safety of patients. The diet was modified, increasing the proportion of natural regulatory bioactive compounds.

## 2. Material and Methods

### 2.1. Study Group

This study was conducted at the Sonomed Medical Centre in Szczecin. Sixty obese female patients were observed after balloon insertion into the stomach using the Bioenteric Intragastric Balloon (BIB) method. All patients were diagnosed with hypertension. The follow-up time was 1 year. Measurements were carried out immediately before the bariatric surgery and at the end of the follow-up period. Ethical approval was obtained from the Bioethics Committee of the Regional Chamber of Physicians in Szczecin on 6 May 2015 (OIL-Sz/Mf/KB/452/06/05/2015). 

A body composition analysis was performed, using a body composition analyser, by the bioimpedance analysis (BIA) method. The IOI 353 analyser was CEO 123 certified and complied with the MDD 93/42/EEC medical device directive. The mean age of the women was 40.0. The mean BMI was 37.2 ± 3.5 kg/m^2^. All patients had a history of numerous unsuccessful attempts at weight reduction by conservative methods, i.e., diet. The patients were under the care of a dietician and a psychologist.

#### Division of the Study Group and Dietary Characteristics

Patients were divided into the following groups (20 each):

Group I—control, not following dietary recommendations;
Group II—following dietary recommendations developed by American specialists from the University of Nevada School of Medicine;
Group III—using proprietary modifications of the US recommendations.

The diet increased the proportion of bioflavonoids, *n*-3 EFAs, and vitamins A, E, and C. Thus, 20 mL of flaxseed oil was introduced into the daily diet of patients in Group III as the main source of *n*-3 EFAs. The patients added the selected oil to soups after cooking, without heat treatment, or as an addition to salads or hummus. Dietary ingredients were selected to increase the anti-inflammatory effect. In addition, patients in this group took a synbiotic every other month. A chemical analysis of the antioxidant properties of the proprietary diet was performed: the antioxidant capacity TEAC (Trolox Equivalent Antioxidant Capacity) was determined according to Re et al. [9], the FRAP (Ferric Reducing Antioxidant Power) capacity was established according to Benzie and Strain [10], and the total polyphenol content was measured according to Cheung et al. [11]. In addition, fatty acid composition and content were assessed, and a sensory evaluation of the proposed diet was carried out [4]. It was assumed that an appropriate combination of bioactive components would confer antioxidant properties to the diet. Groups I and II had similar proportions of macronutrients. The protein content of the diet was 80–85 g/day, whereas that of total carbohydrates was 70 g/day and total fat 42 g/day. The caloric value of the diets at month 1 was about 400 kcal, at month 3 about 600–800 kcal, and at month 5–12 about 1200 kcal.

Patients in Group III supplemented their diet with the addition of 20 mL of flaxseed oil and smoothies (twice a day) high in antioxidants and probiotics. They also took probiotics once a day every other month. 

A 5-point sensory analysis was carried out according to PN-ISO 4121:1998 [12] for unrefined Budwig flaxseed oil (‘LenVitol’ Olfarm Sp.z.o.o, Pietrzykowice), evening primrose oil (‘Wiesiołek’ Olfarm Sp.z.o.o., Pietrzykowice), and Icelandic cod-liver oil (‘LYSIHF’ Reykjavik, Iceland). The discriminants assessed during the evaluation were colour, taste, and odour. The evaluation was carried out at the sensory laboratory of the West Pomeranian University of Technology in Szczecin. The sensory profile analysis of taste and colour was carried out according to PN-ISO 11036:1999 [13], PN-ISO 6658:1998 [14], and PN-ISO 6564:1999 [15]. Sensory evaluation was carried out by a team of 10 assessors trained in oil quality analysis.

The following rating scale was used:

- 5 points—very good;
- 4 points—good;
- 3 points—satisfactory;
- 2 points—barely satisfactory;
- 1 point—unsatisfactory.

The sensory scores were calculated based on the average of the determinants analysed (Table 1).

Based on the analyses, the following composition of fatty acids was determined in the diets of patients from Group III: SFA 26.95%, MUFA 44.98%, *n*-3 PUFA 9.87%, *n*-6 PUFA 18.20, (*n*-6)/(*n*-3) PUFA 1.84, and fat content 8.4%.

Moreover, in food ratios, the composition of fatty acids was determined (Table 2) according to AOAC 2004, (Aa 9-86). The methyl esters of fatty acids were analysed with gas chromatography (AOAC 991.39) using gas chromatography–mass spectrometry (GC-MS). The conditions of the analysis were the following: column SP^TM^-2560, 100× 0.25 cm^3^/min, split 1:50, injector temperature of 220 °C, oven temperature initially at 140 °C, then temperature growth from 140 °C to 240 °C/4 min, reaching a final temperature of 240 °C/10 min.

The cocktails, which were the basis of the diet, contained many more bioactive compounds than the meals of the standard diet. The smoothies were based on vegetables and fruits such as pomegranate, raspberry, currant, orange, apple, spinach, kale, parsley, ginger, beetroot, and pumpkin, and flaxseed was also added. The daily intake of antioxidant components, depending on the type of smoothie consumed, was 5479–8414 µMTE/day in terms of antioxidant capacity (TEAC), 1642–4896 µM TE/day for the reducing capacity, and 1.2–2.2 g catechin equivalents/day for the total polyphenol content [4].

### 2.2. Immunoenzymatic Assays

An immunoenzymatic ELISA technique was used to determine serum prostacyclin, angiotensin, plasminogen, and isoprostane. Absorbance was read at 450 nm ± 2 nm using an ELx808 multichannel ELISA reader (BIO-TEK Instruments, Inc., Winooski, VT, USA). The KC Junior for the Windows calculator programme from BIO-TEK Instruments, Inc., USA, was used to develop calibration curves and calculate the concentration values of the test parameters. The following commercial kits were used for this study:

- Prostacyclin (catalogue no. 515211, Cayman Chemical Company, Ann Arbor, MI, USA), with a method accuracy of ±1% ± 0.010 absorbance and a measurement range of 1.6–1000 pg/mL;
- Angiotensin (catalogue no. E0797h, EIAab Science Co., Wuhan, China), with a method accuracy of ±1% ± 0.010 absorbance and a measurement range of 15.6–1000 pg/mL;
- Plasminogen (catalogue no. E0532h, EIAab Science Co, Wuhan, China), with a method accuracy of ±1% ± 0.010 absorbance and a measurement range of 0.156–10 ng/mL;
- Isoprostane (catalogue no. 516351, Cayman Chemical Company, Ann Arbor, MI, USA), with a method accuracy of ±1% ± 0.010 absorbance and a measurement range of 0.8–500 pg/mL.

### 2.3. Blood Rheological Measurements 

Rheological tests were performed using an AR2000 ex rotational rheometer from TA Instruments (New Castle, DE, USA). The tests were carried out on whole blood at 32 °C using the plate-to-plate method with a diameter of 40 mm, at a constant shear rate of 1 [1/s], to determine apparent viscosity η.

### 2.4. Measurement of Triglyceride Levels and Blood Pressure

In all patient groups, serum triglyceride concentrations were determined in the collected blood. TG determination was performed using a colourimetric method. These tests were performed before the start of treatment and after the weight loss process. Analyses were performed in accredited diagnostic laboratories. Blood pressure was also measured in accordance with the current recommendations of the Polish Society of Hypertension.

### 2.5. Statistical Analysis 

Statistica 13 from Statsoft was used for the calculations. To demonstrate statistically significant differences in concentrations between groups, the non-parametric Wilcoxon paired-rank order test, a non-parametric alternative to Student’s *t*-test for dependent samples, was used.

## 3. Results 

All patients had a history of multiple unsuccessful attempts at weight loss with conservative methods, including dieting. The standard diet used in Group II followed the recommendations of the Polish Society for the Treatment of Obesity regarding the nutrition of people after bariatric surgery [16]. The diet for Group III was antioxidant and anti-inflammatory [4]. At the baseline after treatment, the caloric value of all diets at month 1 was about 400 kcal, while, at month 3, it was about 600–800 kcal and, at months 5–12, about 1200 kcal. A diet of about 1000 kcal had the following values: protein, 80–85 g/day; total carbohydrates, 70 g/day; and total fat, 42 g/day. The *n*-6/*n*-3 value was 1.84. The diet of Group III contained two meals with high antioxidant potential [4]. The caloric value of the diets at month 1 was about 400 kcal, at month 3 about 600–800 kcal, and at months 5–12 about 1200 kcal. Patients in Group III took a synbiotic once a day, every other month. The total number of colony-forming units (CFUs) was 1 × 109 CFUs. 

All measurements were performed in duplicate, at the beginning and at the end of the observation period. Using bioelectrical impedance measurements, the effects of fat reduction were compared. 

**Figure 1:**
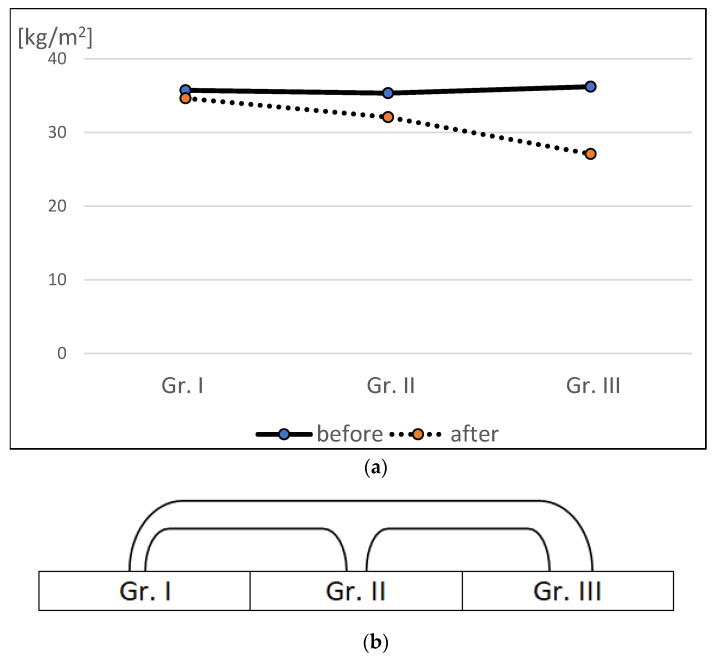
(**a**) BMI values of patients in three groups before and after BIB surgery and weight loss diets. (**b**) Incidence of statistically significant differences between the obtained BMI values of patients in the three groups after BIB surgery and weight loss diets.

Patients adhering to a diet rich in bioflavonoids, vitamins, minerals, *n*-3 fatty acids, and synbiotics achieved the highest weight reduction from an average of 98.5 kg to 70.0 kg, compared to Group II, from 95.0 kg to 80.0 kg, and Group I, from 89.5 kg to 83.4 kg. Group III achieved the highest reduction in body fat mass and visceral fat area, with the lowest loss of muscle mass and BMI (Table 3, Figure 1). The effects of the body composition analysis in Group III are shown in Table 1 and Figure 1. Statistically significant differences were found between Groups I and II, I and III, and II and III, at *p* < 0.05.

**Figure 2:**
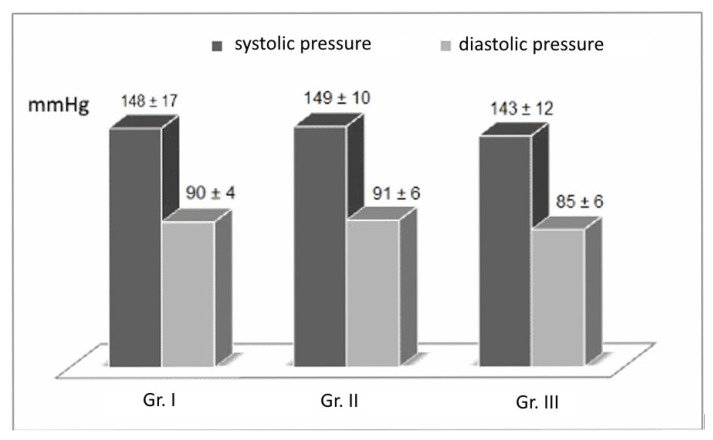
Blood pressure values of patients in the three groups before BIB treatment. Group I—control; Group II—receiving the standard diet; and Group III—receiving the modified diet.

**Figure 3:**
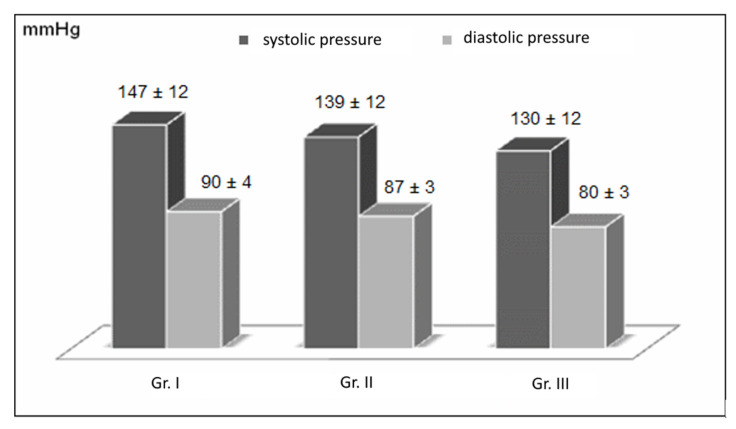
Blood pressure values of patients in the three groups after the BIB procedure and weight loss diets.

In Group II, the blood pressure values before surgery were 149 ± 10/91 ± 6 mmHg and decreased to 139 ± 10/87 ± 2 mmHg after surgery. In Group III, the pressure values before surgery were 143 ± 10/85 ± 5 mmHg and decreased to 130 ± 10/80 ± 3 mmHg after surgery. The changes in systolic and diastolic blood pressure values in Group II, obtained after the diet-assisted BIB procedure, can be considered high but normal. The systolic and diastolic blood pressure values in Group II after the diet-assisted BIB procedure can be considered high but below the threshold for hypertension. The mean systolic and diastolic blood pressures of the patients before and after the treatment are shown in Figure 2 and Figure 3.

**Figure 4:**
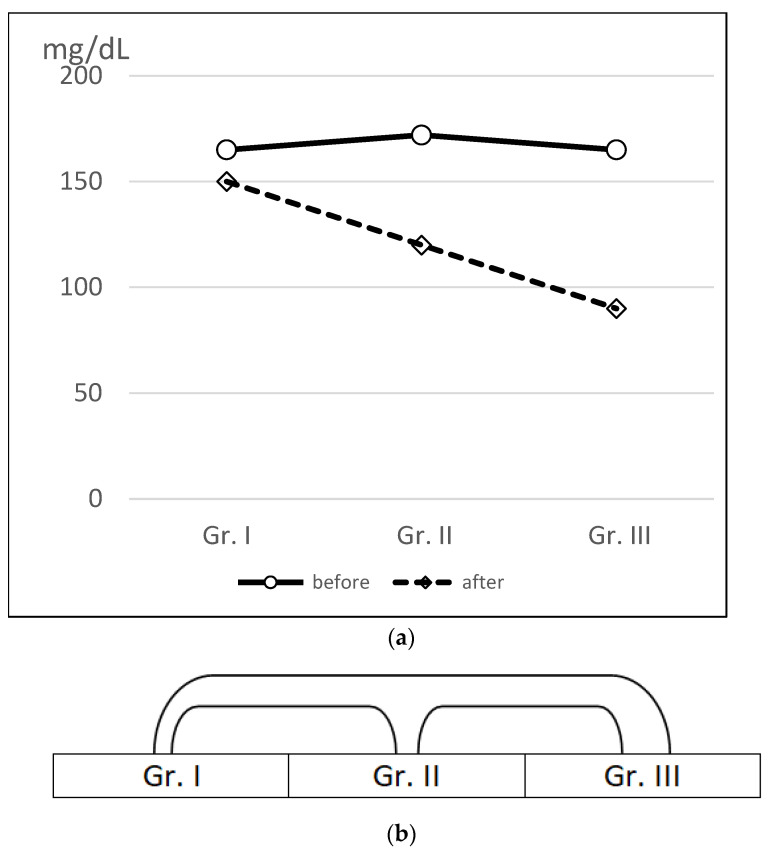
(**a**) Mean triglyceride levels of patients in the three groups before and after BIB surgery and weight loss diets. (**b**) Incidence of statistically significant differences between the achieved triglyceride levels of patients in the three groups after BIB surgery and weight loss diets.

According to the standards of the Polish Society of Cardiology, normal triglyceride levels are 35–150 mg/dL (Polish Society of Lipidology 2016) [17]. The triglyceride levels in patients in Group I, before BIB, were 165 mg/dL and, after the procedure, 150 mg/dL. In Group II patients, following international dietary recommendations, the triglyceride levels before BIB were 172 mg/dL and, after the procedure, 120 mg/dL. In Group III, in patients following the authors’ diet, the triglyceride level before BIB was 165 mg/dL and, after the procedure, 90 mg/dL (Figure 4). Statistically significant differences were found between Groups I and II, I and III, and II and III, at *p* < 0.05.

A non-parametric Wilcoxon paired-rank order test was used, with an assumption of *p* < 0.05, for the results of triglyceride concentrations, comparing data before weight loss to data after treatment. Group I showed no statistically significant differences. Groups II and III showed statistically significant differences in triglyceride concentrations.

Isoprostane concentrations were determined by immunoenzymatic assay, using the ELISA method, and the mean values obtained are shown in Table 4. Lower values of isoprostane levels were obtained in patients on the experimental diet. The determination of isoprostanes provides a reliable and sensitive indicator of the lipid peroxidation metabolism in vivo, allowing the estimation of the contribution of free radicals to the pathophysiology of many diseases. The results obtained may be a marker of reduced free radical processes in the body associated with the pathogenesis of many metabolic diseases associated with obesity.

**Figure 5:**
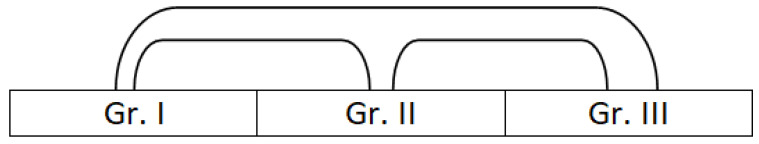
To assess the occurrence of statistically significant differences between the obtained concentrations of isoprostanes, prostacyclins, plasminogen, and angiotensinogen in patients in the three groups after BIB surgery and the weight loss diets.

Using Wilcoxon’s non-parametric paired-rank order test, statistically significant differences were shown between Groups I and II, I and III, and II and III (Figure 5) at *p* < 0.05. There was a significant indication of differences between the results obtained for Groups II and III. It can therefore be assumed that antioxidant components taken systematically significantly inhibit free radical reactions.

Prostacyclin concentrations were determined with an immunoenzymatic assay, using the ELISA method. The mean values obtained are shown in Table 5. In our study, after one year of follow-up, we found higher values of serum prostacyclin in patients from Groups II and III treated with a diet after the BIB procedure. For Group III patients receiving a modified diet, the effect was better because the support was a diet with functional characteristics. The components of the diet that had been modified with functional foods could positively influence the results regarding prostacyclin concentrations in comparisons between Group II and Group III patients. Using Wilcoxon’s non-parametric paired-rank order test, statistically significant differences were shown between Groups I and II, I and III, and II and III at *p* < 0.05, after the patients had completed their weight loss (Figure 5). There was a statistically significant increase in prostacyclin levels in patients who had reduced their body weight. Excess body fat in obesity highly correlates with the development of atherosclerosis. The components of the diet that had been modified with functional foods may have favourably influenced the results of PGI2 concentrations in comparisons between Groups II and III.

Plasminogen concentrations were determined by immunoenzymatic assay, using the ELISA method. The mean values obtained are shown in Table 6. In the case of Group I, the effect of a small but unfavourable increase in plasminogen was demonstrated. There were decreases in the values of serum plasminogen concentrations in Group II and Group III patients treated with a diet after BIB treatment. The effect was stronger in Group III patients receiving a modified diet. The components of the modified diet containing functional foods may have favourably influenced the results regarding plasminogen concentrations in comparisons between Group II and III patients. Using Wilcoxon’s non-parametric paired-rank order test, statistically significant differences were shown between Groups I and II, Groups I and III, and Groups II and III at *p* < 0.05, after the patients had completed their weight loss (Figure 5).

Angiotensinogen concentrations were determined by immunoenzymatic assay, using the ELISA method. The mean values obtained are shown in Table 7. In all patients in Group III, there were decreases in angiotensinogen concentrations after one year of follow-up. The significant decreases in the values obtained in Groups II and III were due to the reduction diet. In Group III, the strongest reduction effects were seen after a diet rich in antioxidants. The results of the comparison of statistically significant differences between the groups are shown in Figure 5.

Since the concentrations of isoprostanes, prostacyclins, plasminogen, and angiotensinogen in all the groups studied showed statistically significant differences, they are presented jointly and graphically in 5. As is well known, excess body fat in obesity promotes the development of atherosclerosis. The components of the diet modified with functional foods may have favourably modified the concentrations of the parameters studied in comparisons between Groups II and III. 

In the absence of significant changes in Group I, Table 8 compares the results of the final concentrations of the studied parameters for Groups II and III only.

Among the haemorheological properties used in the diagnosis of cardiovascular disease, the outstanding parameter is blood viscosity. The viscosity value is mainly influenced by the morphotic elements of the blood and its properties, in addition to changes in temperature and blood flow velocity [18].

**Figure 6:**
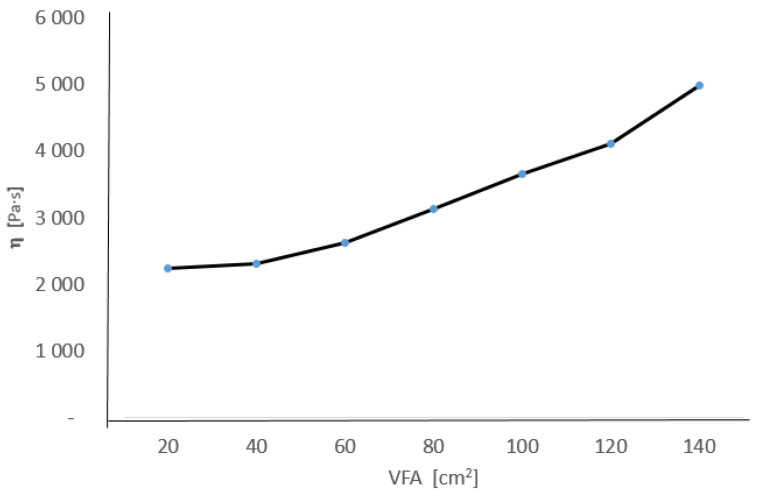
Dependence of blood viscosity on VFA for women (*n*-60).

As VFA (visceral fat area) values depend on the structure of one’s diet, an attempt was made to demonstrate the dependence of viscosity on this parameter (Figure 6). In all patients, lower whole-blood apparent viscosity values were found as the VFA values decreased.

The results are illustrated in the graph below.

## 4. Discussion

We hypothesised that natural biofunctional food ingredients would show potential to modulate many metabolic responses. By creating a diet enriched with bioactive ingredients, a higher reduction in visceral adipose tissue and the regulation of procoagulant parameters were expected. Unfortunately, not all patients showed willingness to cooperate. Patients who did not follow the dietary recommendations formed the control group. The most effective average weight loss was observed in Group III patients following the authors’ diet, rich in bioflavonoids, vitamins, minerals, *n*-3 acids, and synbiotics. There was a reduction in visceral fat in Group II and a stronger reduction in Group III. Patients in Group I, with no diet, did not achieve weight loss. Visceral fat highly correlates with vascular problems [19]. Group III, whose patients received *n*-3/*n*-6 fatty acids in the ratio (*n*-6)/(*n*-3) PUFA = 1.84, showed statistically significant differences in the triglyceride concentrations after the diet period.

As shown in our study, normalisation of blood pressure occurred in the group following the authors’ dietary recommendations. Compared to the control group, statistically significant differences were obtained in Group II and Group III. Despite the application of BIB, persistently high systolic and diastolic blood pressure values were indicated in patients who did not follow the dietary recommendations and did not reduce their body weight (Group I). It can be stated that dietary intervention is necessary. Appropriate dietary components not only support a reduction in adipose tissue but also regulate blood pressure values in patients [20,21]. The development of obesity is accompanied by increased inflammatory activation in adipose tissue.

Increased levels of inflammatory markers are found in obese patients [22,23]. Analyses of isoprostanes are used to monitor oxidative stress levels and eliminate disease factors such as arteriosclerosis, insulin-dependent diabetes, hypercholesterolaemia, obesity, neurodegenerative diseases, asthma, or pneumonia [24,25,26,27]. Chronic platelet activation, that is, the increased synthesis of pro-aggregating isoprostanes as a result of increased oxidative stress, has been observed in obese individuals [28,29]. Weight loss decreased the synthesis of pro-aggregating isoprostanes. 8-iso-PGF2α has been shown to stimulate smooth-muscle contraction in the blood vessel wall of the brain, heart, and kidneys. Under conditions of oxidative stress, 8-iso-PGF2α may increase the risk of embolic complications in patients with cardiovascular disease, including ischaemic stroke or myocardial infarction [30]. Numerous studies have shown significantly higher isoprostane concentrations in obese individuals compared to control groups with normal BMI values. A positive correlation with waist circumference and visceral fatness has been shown [31,32]. Obesity is accompanied by oxidative stress. Metabolic diseases exacerbate lipid peroxidation, increasing isoprostane concentrations. It is known that some dietary components, such as vitamins, plant pigments, bioflavonoids, tannins, fatty acids, and other compounds belonging to the phytamine group, are bioactive compounds which were intentionally increased in our diet. A significant decrease in serum isprostane levels was observed in patients with inflammatory diseases who received higher doses of anthocyanins, derived from berries, strawberries, currants, and blueberries, through their diet [33,34,35]. Oxidative stress is characterised by a lack of capacity of the body’s natural antioxidant defence mechanisms. Therefore, the structure of the designed diet with antioxidant features was not random. In the group of patients receiving the author’s diet, enriched with flavonoids, vitamins, and *n*-3 fatty acids, the obtained values of blood isoprostane concentrations were the lowest compared to the results of serum isoprostane concentrations before balloon implantation and weight loss surgery. When comparing the isoprostane concentrations before and after balloon removal, statistically significant differences were obtained between Groups I and II, I and III, and II and III. No changes in isoprostane concentrations after the follow-up period were seen in the control group of patients not adhering to dietary recommendations. In Group II, the isoprostane concentrations decreased, but this was strongest in Group III. 

The favourable *n*-6/*n*-3 EFA ratio was 4:1. Arachidonic acid metabolism leads to the formation of prostacyclin PGI2. It is used in the prevention of atherosclerosis. It exhibits anticoagulant effects, dilates arteries, prevents platelet aggregation, and dissolves pre-formed thrombi. In addition, its immunomodulatory and anti-inflammatory properties have also been discovered [36]. Visceral obesity promotes thrombus formation and induces endothelial dysfunction. Obese patients show an impaired protective response to nitric oxide and prostacyclin [37]. In obese patients, there is a reduced ability of prostacyclins to activate cAMP synthesis and prevent platelet aggregation [38]. A high correlation between the presence of hypertension and low serum prostacyclin levels has been indicated [39]. In our study, in Group III patients, there was a significant reduction in visceral adipose tissue, an improvement in blood pressure values and TG levels, and elevated prostacyclin levels compared to the results obtained in Group II and I patients. The improvement in test parameters may indicate a reduction in the patients’ risk of atherosclerosis. Analysing the results of the obtained prostacyclin concentrations, statistically significant differences were indicated between Groups I and II, I and III, and, significantly, II and III.

The endocrine–enzymatic system, AAR (renin–angiotensin–aldosterone), is responsible for the regulation of blood pressure and water–electrolyte balance. It acts systemically in the bloodstream and locally, e.g., in blood vessel walls or the myocardium [40]. Clinical trials targeting angiotensinogen in the treatment of hypertension and heart failure are ongoing. Higher levels of angiotensinogen correlate strongly with the incidence of obesity and hypertension, especially in women [41,42]. As adipocytes are a local source of angiotensinogen synthesis [43], their concentration as angiotensin prohormones was determined in our study. Increased adipose tissue contributes to the systemic up-regulation of the AAR system. This promotes inflammation, lipogenesis, and reactive oxygen species production and impairs insulin signalling. The increase in blood concentrations of AAR components mediated by adipocytes is the link between hypertension and inflammation in obesity. This is an example of a vicious circle. In our own observation, initially, in the presence of obesity, all patients had high levels of angiotensinogen. After BIB and a diet based on natural regulating ingredients, a significant decrease in the angiotensin prohormone was found. An association of high-sugar and high-fat diets with the components of the AAR has been established [44]. In contrast, regulating the proportion of polyunsaturated acids (PUFAs) in one’s diet positively regulates blood pressure. Gamma linolenic acid attenuates the development of hypertension. It lowers the plasma aldosterone levels and decreases the density and affinity of AT1 in the adrenal glands [45]. A diet rich in omega-3, compared to a diet poor in this acid, reduces hypertension through the modulation of the RAS [46]. Group III, in which patients received *n*-3/*n*-6 fatty acids at a ratio of (*n*-6)/(*n*-3) PUFA = 1.84, showed beneficial changes in angiotensinogen regulation.

The potential involvement of the overproduction of plasminogen activator inhibitor type 1 in the pathogenesis of increased prothrombotic activity in obese individuals has been analysed. It has been shown that the adipocytes of an obese person produce 2-fold more PAI-1 than in a lean person. PAI-1 is also identified with the development of atherosclerosis and coronary thrombosis accompanying atherosclerotic plaque rupture [47]. PAI-1 is considered an acute-phase protein. Its regulation is influenced by pro-inflammatory factors (IL-1, TNFα) but also angiotensin II and IV [48,49]. Elevated levels of PAI-1 have been shown to highly correlate with coronary artery disease and myocardial infarction [50,51]. In our study, patients receiving a diet rich in antioxidant compounds showed the lowest plasminogen concentrations.

Haemorheological properties are used in the diagnosis of cardiovascular diseases. A prominent parameter is blood viscosity. The viscosity value is mainly influenced by the morphotic elements of the blood and its properties, in addition to changes in temperature and blood flow velocity [18]. 

Severe haemorheological disorders are diagnosed in the presence of obesity. Obese patients show increased viscosity values and decreased rheological values for blood flow velocity, especially through the capillaries. This results in increased peripheral resistance and, thus, the need for haemodynamic adaptation.

This study showed that, after a diet rich in antioxidants, the surface area of visceral fat decreases, which had a direct effect on the decrease in blood viscosity. Adipocytes are connected to capillaries. Their excess forces the synthesis of newly formed vessels. This leads to an increase in the vascular bed area. Vascular reactivity is altered. Under typical stimuli, they do not contract or contract insufficiently [2]. This results in higher systolic blood pressure values, increased left ventricular contractile force, overload, and hypertrophy. The higher the BMI values, the higher the circulating blood volume in the body. At the same time, the haematocrit index highly correlates with increased body fat [2]. Due to impaired blood flow through adipose tissue, a weaker organ blood flow is observed [52]. The increase in venous pressure probably depends on the slower outflow of blood from the large veins and the right half of the heart. Oxygen’s concentration in venous blood is markedly reduced [52]. In our study, the dependence of blood viscosity on the visceral fat area (VFA) was indicated. A decrease in the VFA values was obtained, thereby lowering the apparent blood viscosity values, regulating the prothrombotic parameters, and improving the haemorheological properties of the patients’ blood.

The study shows that the BIB procedure, followed by dietary support, is an effective method of weight reduction. By modifying the diet, enriching it with natural bioactive ingredients, significantly better fat reduction results and favourable values for cardiovascular parameters were achieved.

## 5. Conclusions

At the beginning of follow-up, obese patients showed unfavourable values for triglycerides, blood pressure, and prothrombotic indices. Using a functional diet, a significant reduction in visceral fat was achieved. A decrease in VFA was shown to reduce whole-blood viscosity. Furthermore, this study showed a significant effect of bioactive components on prothrombotic parameters in obese patients. The improvement in the parameters analysed may indicate a reduction in the risk of atherosclerosis in these patients.

## Figures and Tables

**Table 1:** Sensory analysis of oils.

Oil	Discriminants				
	Appearance	Colour	Taste	Smell	Total Score
Linseed oil	4.50	4.20	1.80	2.80	3.33
Evening primrose oil	4.00	4.00	1.25	1.80	2.76
Icelandic cod-liver oil	3.80	4.00	1.00	1.00	2.45

**Table 2:** Fatty acid composition analysis.

Systematic Name of Fatty Acid	Lipid Number	Fatty Acid Content [%]	SD
Tetradecanoic	C14:0	2.862	0.09
Pentadecanoic	C15:0	0.163	0.02
Hexadecanoic	C16:0	19.986	0.15
	C16:1	4.339	0.11
Octadecanoic	C18:0	3.94	0.09
	C18:1	38.156	0.21
Octadecadienoic	C18:2 *n*-6	15.63	0.18
	C18:3 *n*-3	6.65	0.12
Octatetraenoic	C18:4 *n*-3	0.777	0.02
Docosanoic	C22:1	2.382	0.08
Eikosatetraenoic	C20:4 *n*-6	2.571	0.03
	C20:4 *n*-3	0.10	0.01
Eikosapentaenoic	C20:5 *n*-3	1.10	0.04
Tetracosanoic	C24:1	0.101	0.01
Docosapentaenoic	C22:5 *n*-3	0.20	0.03
Docosahexaenoic	C22:6 *n*-3	1.05	0.07
	SFA	26.95	
	MUFA	44.98	
	*n*-3 PUFA	9.87	
	*n*-6 PUFA	18.20	
	(*n*-6)/(*n*-3) PUFA	1.84	
	Fat content	8.4	0.16

**Table 3:** Analysis of body composition by bioelectrical impedance ($\bar{x}$ ± SD).

	Fat Mass [kg]		Visceral Fat Area [cm^2^]		Muscle Tissue Mass [kg]	
	Before	After	Before	After	Before	After
Gr.1	39.5 ± 3.2	36.6 ± 1.2	140 ± 22.5	132 ± 16.3	40.4 ± 2.0	37.2 ± 1.8
Gr.2	40.2 ± 2.5	31.1 ± 2	149.5 ± 25.7	124.5 ± 12.3	45.3 ± 1.6	39.5 ± 2.2
Gr.3	42.4 ± 1.2	20.1 ± 1.5	139.8 ± 19.9	95.8 ± 8.3	46.1 ± 3.1	39.9 ± 2.0

**Table 4:** Mean values of serum isoprostane levels [pg/mL] in patients in the three groups before and after the BIB procedure and the weight loss diet.

Group I		Group II		Group III	
Before BIB $\left(\bar{x}\pm SD\right)$	After BIB $\left(\bar{x}\pm SD\right)$	Before BIB $\left(\bar{x}\pm SD\right)$	After BIB $\left(\bar{x}\pm SD\right)$	Before BIB $\left(\bar{x}\pm SD\right)$	After BIB $\left(\bar{x}\pm SD\right)$
145.8 ± 16.8	148.3 ± 21.1	137.4 ± 20.5	110 ± 20.8	143.3 ± 23.8	94.3 ± 23.2

**Table 5:** Mean prostacyclin levels [pg/mL] in the serum of patients in the three groups before and after BIB surgery and relevant weight loss diets.

Group I		Group II		Group III	
Before BIB $\left(\bar{x}\pm SD\right)$	After BIB $\left(\bar{x}\pm SD\right)$	Before BIB $\left(\bar{x}\pm SD\right)$	After BIB $\left(\bar{x}\pm SD\right)$	Before BIB $\left(\bar{x}\pm SD\right)$	After BIB $\left(\bar{x}\pm SD\right)$
9.51 ± 1.09	9.84 ± 1.31	9.39 ± 1.22	16.93 ± 1.66	8.58 ± 1.39	21.81 ± 1.37

**Table 6:** Mean plasminogen levels [ng/mL] in the serum of patients in the three groups before and after BIB surgery and relevant weight loss diets.

Group I		Group II		Group III	
Before BIB $\left(\bar{x}\pm SD\right)$	After BIB $\left(\bar{x}\pm SD\right)$	Before BIB $\left(\bar{x}\pm SD\right)$	After BIB $\left(\bar{x}\pm SD\right)$	Before BIB $\left(\bar{x}\pm SD\right)$	After BIB $\left(\bar{x}\pm SD\right)$
20.03 ± 0.6	20.59 ± 0.91	19.26 ± 0.53	12.13 ± 0.53	19.29 ± 0.59	9.57 ± 0.59

**Table 7:** Mean values of angiotensinogen [pg/mL] in the serum levels of patients in the three groups before and after BIB surgery and relevant weight loss diets.

Group I		Group II		Group III	
Before BIB $\left(\bar{x}\pm SD\right)$	After BIB $\left(\bar{x}\pm SD\right)$	Before BIB $\left(\bar{x}\pm SD\right)$	After BIB $\left(\bar{x}\pm SD\right)$	Before BIB $\left(\bar{x}\pm SD\right)$	After BIB $\left(\bar{x}\pm SD\right)$
4.16 ± 0.41	3.71 ± 0.48	3.57 ± 0.68	2.88 ± 0.25	3.39 ± 0.41	2.34 ± 0.37

**Table 8:** Comparison of parameter concentrations for Groups II and III after 12 months of observation (*p* < 0.05).

Parameter	II gr (*n*-20)	III gr (*n*-20)
BMI [kg/m^2^]	32.07 ± 5.82	27.07 ± 4.86
VFA [cm^2^]	124.5 ± 12.3	95.8 ± 8.3
Blood pressure [mmHg]	139 ± 12/87 ± 3	130 ± 12/80 ± 3
TG [mg/dL]	120 ± 9.23	90 ± 6.78
Isoprostanes [pg/mL]	110 ± 20.88	94.32 ± 23.27
Plasminogenes [ng/mL]	12.13 ± 0.53	9.57 ± 0.59
Angiotensines [pg/mL]	2.88 ± 0.26	2.34 ± 0.37
Prostacyclines [pg/mL]	16.93 ± 1.66	21.81 ± 1.37

## Data Availability

The original contributions presented in this study are included in the article. Further inquiries can be directed to the corresponding author.
